# Treatment of Cutting Fluid Waste using Activated Carbon Fiber Supported Nanometer Iron as a Heterogeneous Fenton Catalyst

**DOI:** 10.1038/s41598-018-29014-4

**Published:** 2018-07-13

**Authors:** Chunjian Su, Gaohua Cao, Shumei Lou, Rui Wang, Fengru Yuan, Longyun Yang, Qing Wang

**Affiliations:** 1 Department of mechanical design and manufacturing engineering, College of Mechanical and Electronic Engineering, Shandong University of Science and Technology, Qingdao, 266590 China; 2 Department of polymer materials, School of Material Science and Engineering, Shandong University of Science and Technology, Qingdao, 266590 China

## Abstract

Addressing the problem of high chemical oxygen demands (COD) of cutting fluid waste generated in the machining process, its complex composition, and the specific conditions required for the treatment process, a heterogeneous Fenton fibre catalyst (NZVI@ACF) made of nanometer-iron supported on activated carbon fiber using dip-molding was developed. NZVI was homogeneously loaded onto ACF surfaces to form NZVI@ACF, with a specific surface area (S_BET_) of 726.3642 m^2^/g. Using a multistage chemical pretreatment, the NZVI@ACF/H_2_O_2_ system was used to effectively treat cutting fluid waste. The results indicated that the rate of COD removal in the cutting fluid waste liquid pretreated with NZVI@ACF/H_2_O_2_ system was 99.8% when the reactions conditions were optimized to 20 nmol/L H_2_O_2_, 6 g/L NZVI@ACF, total reaction time of 120 min and pH 5. The treated waste solution passed China’s tertiary wastewater discharge standards. NZVI@ACF/H_2_O_2_ demonstrated an excellent catalytic performance compared to the traditional Fenton catalyst, increased the effective pH reaction range and had an adsorption effect on the waste liquid after the reaction.

## Introduction

Mechanical processing using large amounts of cutting fluid results in liquid waste with a high COD and complex composition^1,2^, which is difficult to degrade. This waste is difficult to manage using conventional physical and biological methods^3^. Several studies have been reported describing physical separation^4^, chemical treatment^5^, biodegradation^6,7^ and advanced oxidation methods to manage the waste^8,9^. Of these methods the coagulation-advanced oxidation method is the most efficient and of these the Fenton system is considered to be a promising technology among the advanced oxidation processes (AOPs)^10,11^, because it has a wide range of application and strong anti-interference ability^12^. Hydroxyl radicals is the main product of this system, which is the key to the treatment of organic matter^13^. However, the pH range of the traditional Fenton system is relatively narrow and demanding and usually controlled at around 3^14,15^. A large amount of acid and alkali in actual wastewater treatment are required to regulate pH value and increase the cost^16,17^. Moreover, iron salts^18^ that act as catalysts in the reaction change to iron mud^19^ after reaction, which requires additional separation steps^20^ and easily causes secondary contamination^21^. This limits the use of the Fenton method in wastewater treatment^22,23^.

In contrast, a heterogeneous Fenton system can be used in a broader PH range than the traditional Fenton system^24,25^, where iron ions remain on the surface of the heterogeneous catalyst for reuse^26,27^. The high-performance heterogeneous catalyst can be prepared by loading various iron compounds onto a carrier that has a porous structure^28–30^. Its catalytic performance is stable^31^, and iron dissolution is negligible^32^. Activated carbon fiber (ACFs) can be used as a carrier of heterogeneous Fenton catalyst because its’ characteristics^33,34^: (i) highly developed microporous structure with large adsorption capacity^35^; (ii) ACFs have strong adsorption capacity for non-polar or weak polar organic matters^36^; (iii) ACFs improves the stability and possible reuse of catalyst due to its structural and surface chemical diversity^37^.

Zero-valent iron nanoparticles (NZVI) have been used in a Fenton-like system^38^ and were demonstrated to be a versatile material, capable of reducing/adsorbing anionic inorganic compounds, heavy metals and organics^39^, due to its large specific surface area^40^, high chemical reactivity and capacity to generate active oxygen species in aqueous solutions.

NZVI@ACF was prepared using the properties of ACF and NZVI^41,42^. This study was based on the heterogeneous Fenton system made from NZVI @ACF that had undergone a multi-stage chemical pretreatment to produce a NZVI@ACFS/H_2_O_2_ system. The efficacy of this NZVI@ACF/H_2_O_2_ system compared to the traditional flocculation-Fenton system was studied under different conditions to determine the influence of pH, amount of catalyst, H_2_O_2_ dosage and reaction time. The results indicated a 99.8% reduction in COD in the waste after treatment with the multi-stage chemical pretreated NZVI@ACFS/H_2_O_2_ system, and a greater tolerance of a broader range of pH values, compared to the traditional Fenton reaction. The treated waste was in compliance with China’s tertiary wastewater discharge standards.

## Results and Discussion

### Selection of experimental reagents for first chemical pretreatment

**Figure 1:**
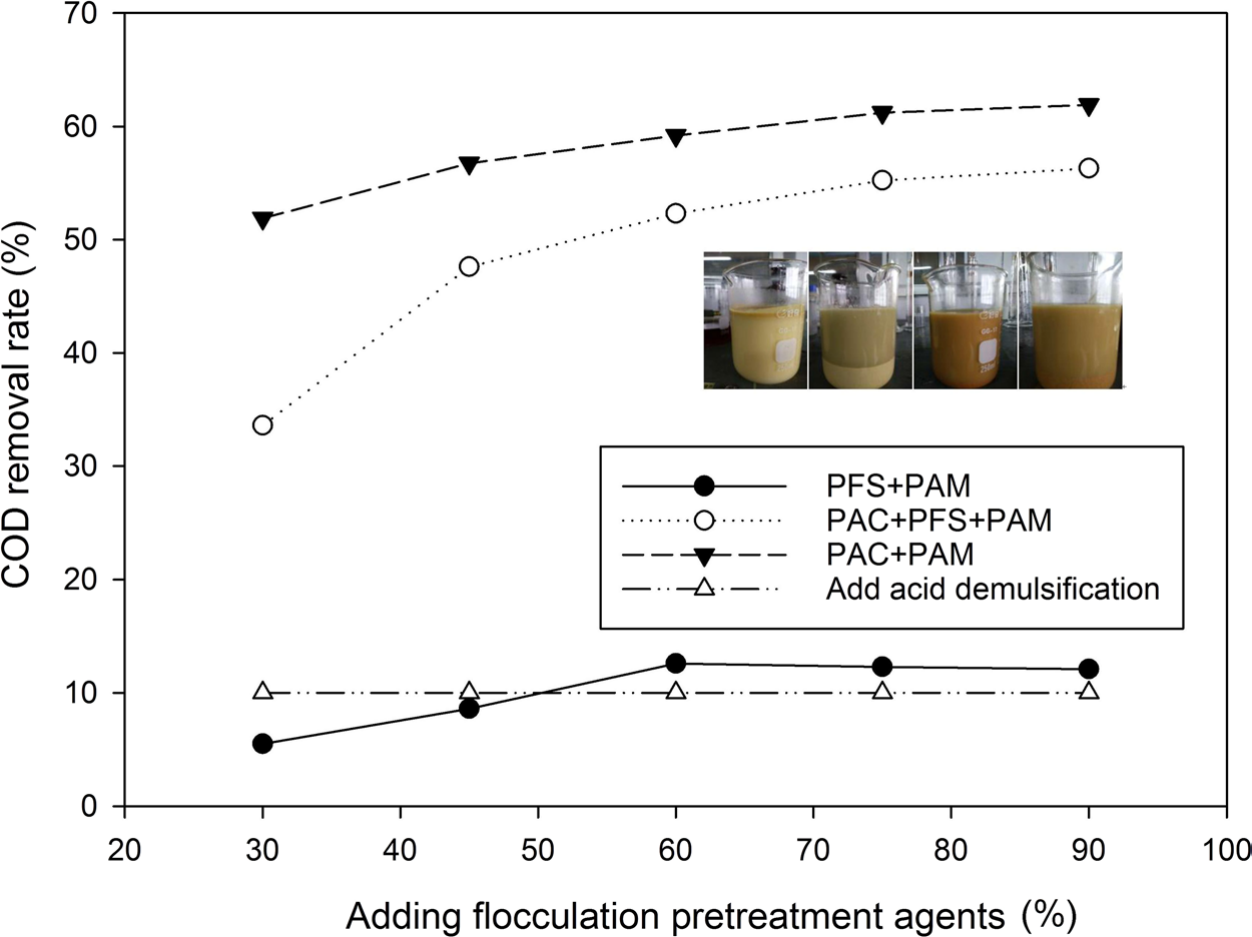
Comparison of the four flocculation pretreatments.

PAC and PFS are positively charged inorganic polymer flocculants that can effectively neutralize the negative charge on the surface of oil droplets, promoting coagulation. PAM is a linear organic polymer used as a macromolecular water treatment flocculent to adsorb suspended particles in water. It links fine particles into large flocs and speeds up sedimentation. The amount of each agent added according to the test method is shown in Table 1. The results of the first chemical pretreatment of NZVI@ACF are shown in Fig. 1.

**Table 1:** Quantity of flocculation pretreatment agents.

Number	10% PAC (mL)	10% PFS (mL)	0.3% PAM (mL)
1	30.0		10.0
2	45.0		10.0
3	60.0		10.0
4	75.0		10.0
5	90.0		10.0
6		30.0	10.0
7		45.0	10.0
8		60.0	10.0
9		75.0	10.0
10		9.0	10.0
11	15.0	15.0	10.0
12	22.5	22.5	10.0
13	30.0	30.0	10.0
14	37.5	37.5	10.0
15	45.0	45.0	10.0

The combination of coagulants was effective in reducing the electrostatic repulsion between the oil droplets and strengthening the separation of oil and water. Some combinations of coagulants could change the charge on the oil droplets, creating re-stabilization or the colloidal coagulant phenomenon, resulting in stable or increasing cutting fluid COD. Analysis of the experimental results suggested that PAC + PAM had the greatest flocculent effect of all the treatments.

### Optimization and results of first flocculation experiment

**Figure 2:**
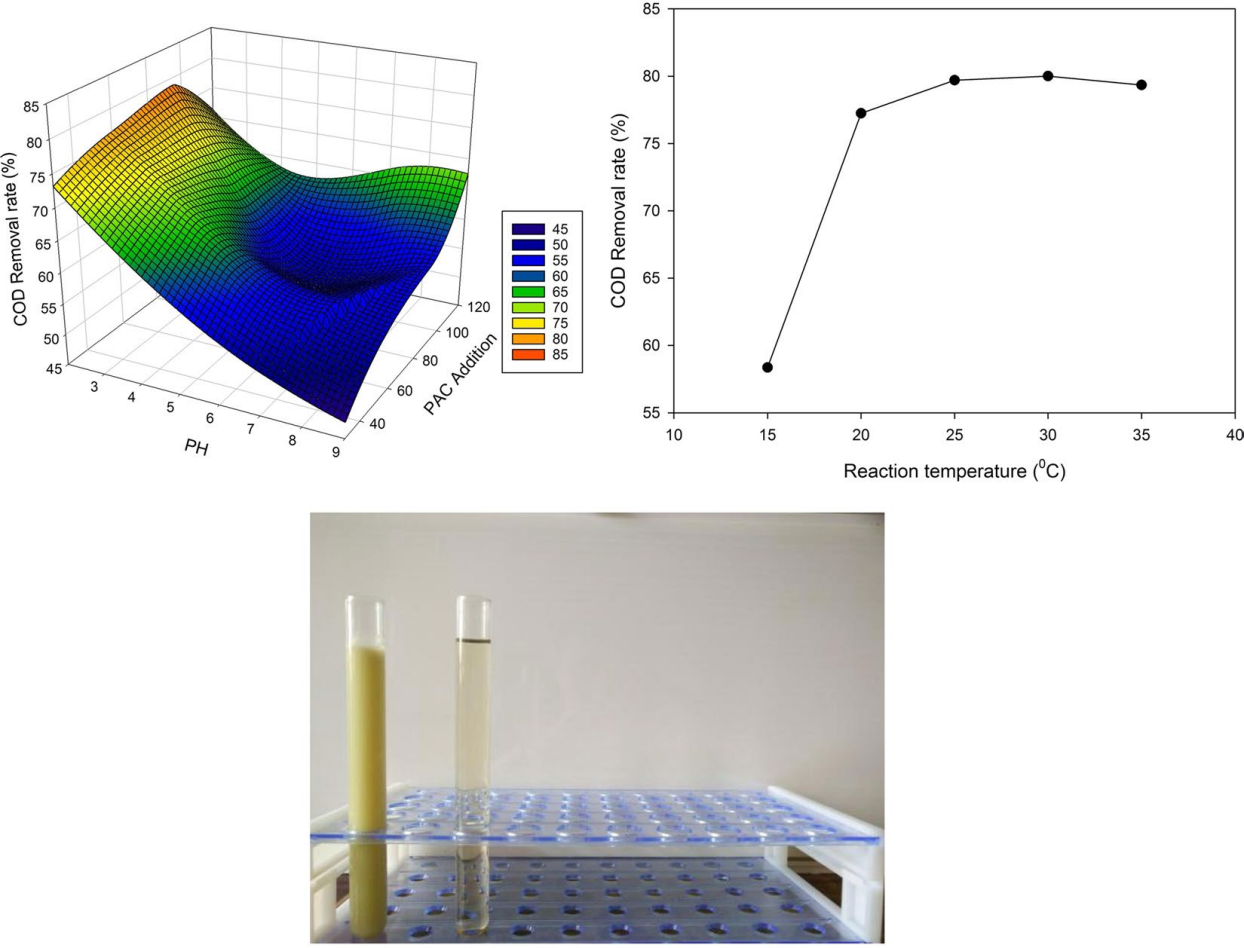
(**a**) The response surface of the effect of pH value with different concentrations of PAC on the rate of COD removal, (**b**) Effect of reaction temperature on the rate of COD removal, (**c**) Comparison of water samples before and after flocculation II.

In a flocculation experiment, the pH value, amount of flocculent added and reaction temperature were the main factors affecting the rate of COD removal. To determine the factors and interactions on the impact of cutting fluid waste treatment a response surface analysis was undertaken. The response surface of the effect of different PAC concentrations and changing pH and reaction temperature on the rate of COD removal are shown in Fig. 2a,b, respectively. The comparison of water samples before and after flocculation is shown in Fig. 2c.

When the slope of the response surface graph is relatively flat within a certain range, little effect on the rate of COD removal from the cutting fluid waste within that range is indicated. In contrast, if the slope of the response surface is very steep, it indicates that the rate of COD removal is sensitive to the factor. Therefore, the optimal conditions for t primary flocculation treatment, as indicated by Fig. 2a,b, were 90 mL PAC and 10 mL PAM at a reaction temperature of 25 °C and reaction time of 5 min. Under these conditions the rate of COD removal was 79%.

### Selection and optimization of experimental conditions for second flocculation

**Figure 3:**
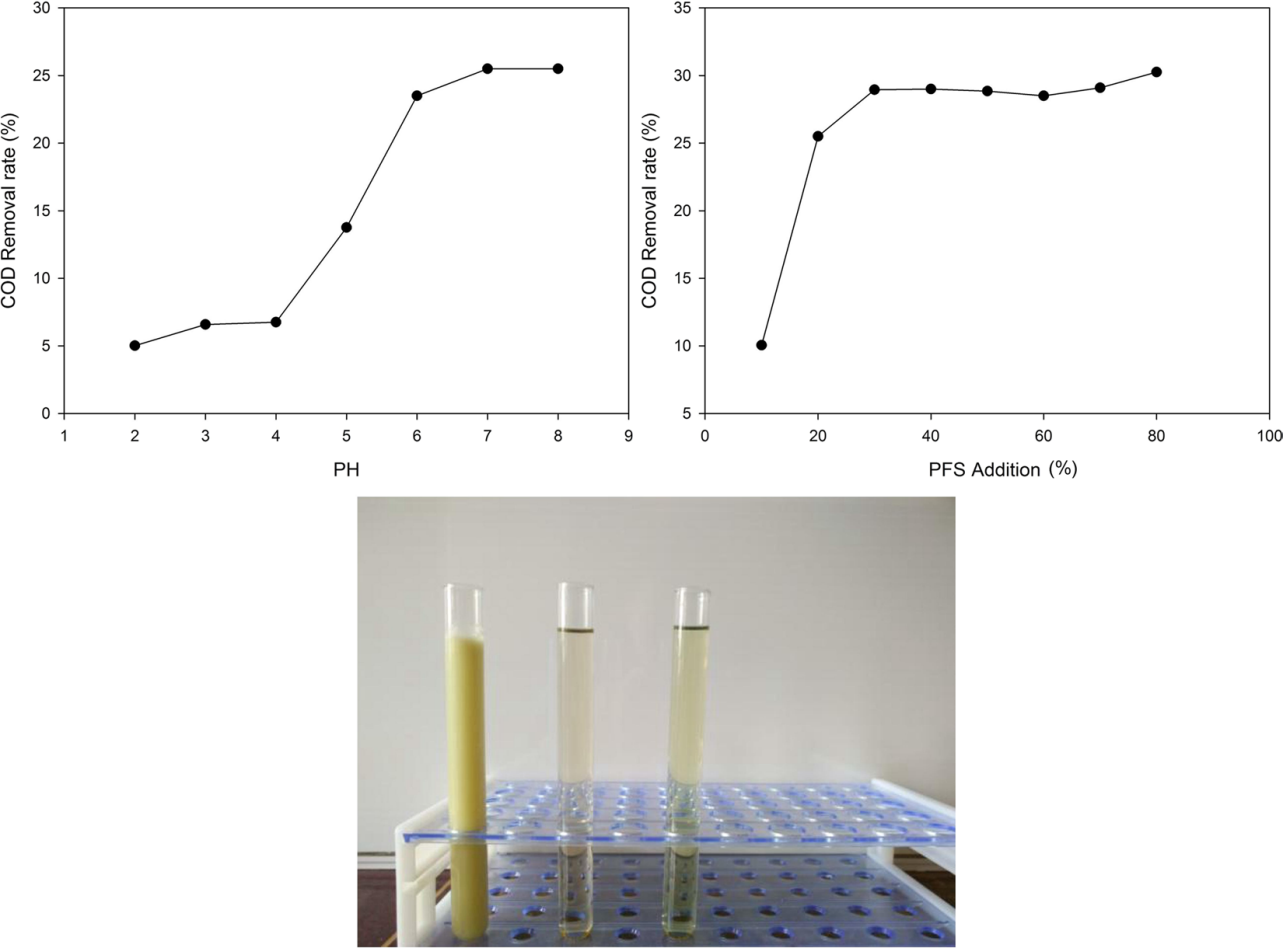
(**a**) Effect of pH on the rate of COD removal, (**b**) Effect of PFS addition on the III removal of COD (pH = 7), (**c**) Visual comparison between the original solution and the solution following primary and secondary flocculation.

Following the primary stage flocculation pretreatment of NZVI@ACFS, the filtrate contained small amount of impurities. The effect of PFS flocculent on the filtered waste was studied. The optimum pH value and amount of PFS added were selected using a single factor experiment (Fig. 3a,b). Visual comparison of the original solution and the solutions following primary and secondary flocculation are shown in Fig. 3c.

The second flocculation was done at pH 7 with a PFS concentration of 30 mL/L. The color of the waste water after secondary flocculation was slightly deepened, due mainly to an increase in Fe^3+^ during the second flocculation process resulting from adjusted pH. The rate of COD removal after the second flocculation compared to the original liquid was 85%.

### Analysis of activated carbon fiber supported nanometer iron characterization results

**Figure 4:**
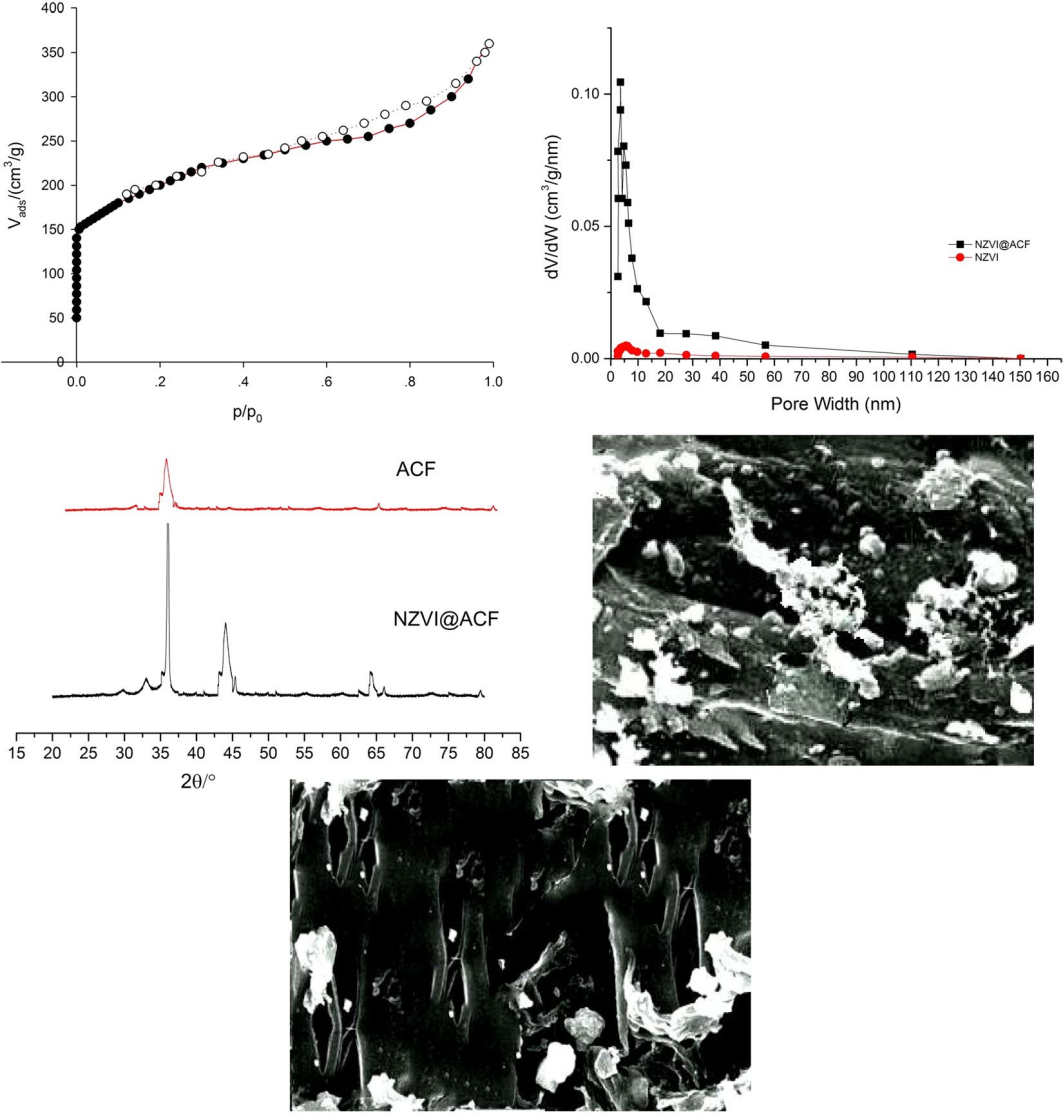
(**a**) N_2_ adsorption/desorption isotherm liner plot of NZVI@ACF catalyst, (**b**) The pore width of NZVI and NZVI@ACF catalyst, (**c**) XRD patterns of ACF and NZVI@ACF, (**d**) SEM images of NZVI@ACF, (**e**) SEM images of NZVI@ACF internal hole.

The nitrogen adsorption isotherm of NZVI@ACF is shown in Fig. 4a. The pore width of NZVI and NZVI@ACF catalyst are shown in Fig. 4b. The BET specific surface area was 726.3642 m^2^/g and the pore volume was 0.7276 mL/g. The micropore area is 608.6172 m^2^/g and the micropore volume is 0.6256 mL/g. The average adsorption mesopore size was 3.78 nm, using the BJH (Barrett-Joiner-Halenda) model. The isotherm was classified using the IUPAC system as a type I, H4 hysteresis loop. It can be seen in Fig. 4a that the adsorption capacity in the low-pressure section was increasing rapidly due to the gas entering the micropores, filling the monolayer with the nitrogen, and filling the multilayers. The strong adsorption trend was due to the presence of more micropores.

A more gradual increase in the high-pressure section indicated that the material was unevenly porous, and the presence of H4 hysteresis loops suggested that holes were created in irregularly stacked layers. A decreased rate of nitrogen adsorption demonstrated that the nanowire was loaded onto the ACF. Therefore, the material obtained from the analysis suggested that NZVI@ACF can be used to adsorb and remove contaminants from waste water, and the ·OH produced by the Fenton reaction can be used to degrade the cutting fluid waste.

If the diffraction pattern and intensity of the test material (NZVI@ACF) and the standard (ACF) are similar, the test material can be considered a standard crystal material. The XRD patterns of ACF and NZVI@ACF are shown in Fig. 4c. Four sharp diffraction peaks at 2θ = 36.5°, 43.5°, 64.5° and 78.5° for NZVI@ACF were observed. The XRD measurement verified that the characteristic peak of the nanometer iron load was at 2θ = 43.5°, and that nanometer iron was successfully attached to the activated carbon fiber. The particle diameter of NZVI@ACF was calculated to be 40 nm, using the Scherrer formula.

Figures 4d,e show SEM images of activated NZVI@ACF. The activated carbon fiber retained the morphological characteristics of the unactivated fiber, with a cylindrical or stick-like shape. In Fig. 4e an increase in the number of holes can be observed, increasing the specific surface area and particulate matter is above load up iron nanoparticles, nanoiron. These images suggest the efficacy of the NZVI@ACF composite.

### Effect of reaction conditions on the treatment of cutting fluid waste using a NZVI@ACF heterogeneous Fenton catalyst

**Figure 5:**
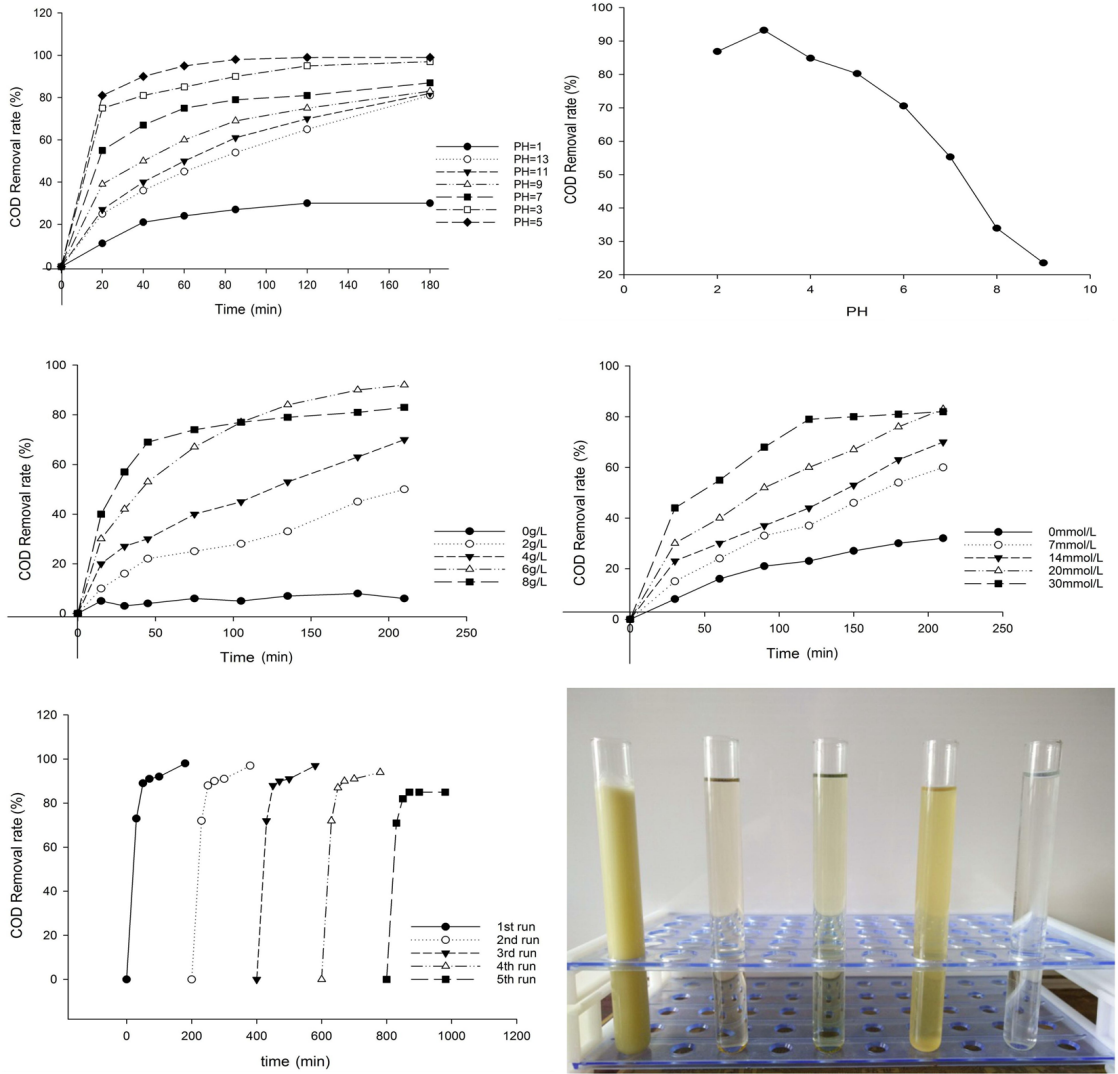
(**a**) Effect of pH on the rate of removal of COD using NZVI@ACF, (**b**) Effect of pH on the rate of removal of COD using the traditional Fenton system, (**c**) Effect of NZVI@ACF addition on the removal of COD, (**d**) Effect of H_2_O_2_ addition on the rate of COD removal, (**e**) Performance of NZVI@ACF over multiple runs, (**f**) Photo of (1) untreated cutting fluid, (2) mixture after primary flocculation, (3) mixture after secondary flocculation, (4) mixture after traditional Fenton treatment, and (5) mixture after heterogeneous Fenton treatment.

One important factor affecting the catalytic ability of the Fenton system is pH. It has a significant influence on catalyst activity and the stability of H_2_O_2_. To investigate the effect of pH, 1 L of waste liquid from the multi-stage flocculation treatment, catalyst (4 g/L) and H_2_O_2_ (20 mmol/L) were used. Figure 5a shows the effect of pH on the rate of removal of COD using the NZVI@ACF system. Figure 5b shows the effect on the rate of removal of COD using the traditional Fenton system.

Figure 5a shows that the NZVI@ACF/H_2_O_2_ system enables degradation across a broad pH range, and Fig. 5b shows that COD removal rate decreased with an increase in pH. Acidic conditions are relatively favorable for the degradation reaction by accelerating surface corrosion of Fe^0^, thereby increasing the number of active sites promoting the formation of Fe^2+^. Increased Fe^2+^ promotes formation of ·OH radicals and increased oxidation capacity. Under alkaline conditions, although the rate of COD removal was reduced, the processing capacity still occurred. Comparing Fig. 5a to Fig. 5b, the greater efficacy of the NZVI@ACF/H_2_O_2_ system over a broader pH range can be seen.

The effect of different concentrations of NZVI@ACF (0, 2, 4, 6, 8 g/L) were compared at pH 5 using cutting fluid waste (1 L), and H_2_O_2_ (20 mmol/L). The results are shown in Fig. 5c. When the dosage of NZVI@ACF was 0 g/L, the effect of H_2_O_2_ on the cutting fluid waste i\was investigated. When the concentration of catalyst was increased, the rate of COD removal also increased. This suggested that NZVI@ACF had a significant effect in the treatment of cutting fluid waste. As the concentration of NZVI@ACF increased, the number of surface active sites increased, which accelerated the rate of decomposition of H_2_O_2_. However, when the concentration of catalyst was 8 g/L, the rate of COD removal plateau at a lower level, below that observed at 6 g/L. This could be due to the catalysis of the production of oxygen from H_2_O_2_ or the catalysis of H_2_O_2_ decomposition from excess ·OH. Both these effects could reduce the rate of COD removal.

Based on previous experiments on cutting fluid waste (1 L) reporting optimum conditions of pH 5 and a NZVI@ACF concentration of 6 g/L, the effect of different H_2_O_2_ concentrations (0, 7, 14, 20 and 30 mmol/L) was investigated. The results are shown in Fig. 5d. As the H_2_O_2_ concentration increased, the rate of COD removal initially increased and subsequently decreased to a stable concentration. This indicated that at a concentration of H_2_O_2_ that were too high or too low, the rate of COD removal was reduced. This could be explained by the small amount of H_2_O_2_ being insufficient ·OH for the reaction to proceed; or in contrast, an excess H_2_O_2_ creates ·OH quenching and decomposition, both of which inhibit the Fenton reaction.

The heterogenous Fenton system has several advantages compared with the traditional Fenton system, in addition to the greater effective pH range. These include the catalyst being able to be recycled and reused multiple times, whilst maintaining performance. The catalytic performance of NZVI@ACF is shown in Fig. 5e.

After five repeats of recycled catalyst, the rate of COD removal reached 82%, demonstrating the good stability of the catalyst. Moreover, the chemical pretreatment did not need to be conducted before each recycling test. The mixture was allowed to stand and then filtered to separate the solid-liquid phases. The catalyst was then rinsed with distilled water and dried for use in the next experiment.

Therefore, the NZVI@ACF catalyst prepared in this experiment had a good catalytic performance and stability, could be recovered simply, and was easy to prepare.

Figure 5f shows that the color of the cutting fluid after the traditional Fenton reaction: Fig. 5f(4) was deeper in color after the multi-stage chemical pretreatment shown in Fig. 5f(2) and f(3), due mainly to the addition of a large amount of FeSO_4_·7H_2_O. FeSO_4_·7H_2_O was oxidized to Fe^3+^ and some Fe^3+^ remained in the step where the pH was adjusted, resulting in a further darkening of color and the secondary contamination. The NZVI@ACF/H_2_O_2_ system did not show this color change. The rate of COD removal in Fig. 5f(5) was 99.8%, and the pollutant content met the national requirements.

### The surface properties of the catalyst before and after the processes

By comparing the FT-IR spectra of the catalyst before and after the reaction, it can be seen that the functional groups on the surface of the catalyst had changed. The band at approximately 3403 cm^−1^ belonged to the vibration of hydroxyl groups. The signal at 1574 cm^−1^ was ascribed to the C=O stretching vibration, the peak at 1060 cm^−1^ was assigned to C-O groups, the signal at 877 cm^−1^ was stronger, indicating that the surface groups of the catalyst increased. The spectra of the NZVI@ACF catalyst before and after degradation did not show significant changes, which confirmed that the catalyst was very stable and could have a good catalytic activity over a long time period.

**Figure 6:**
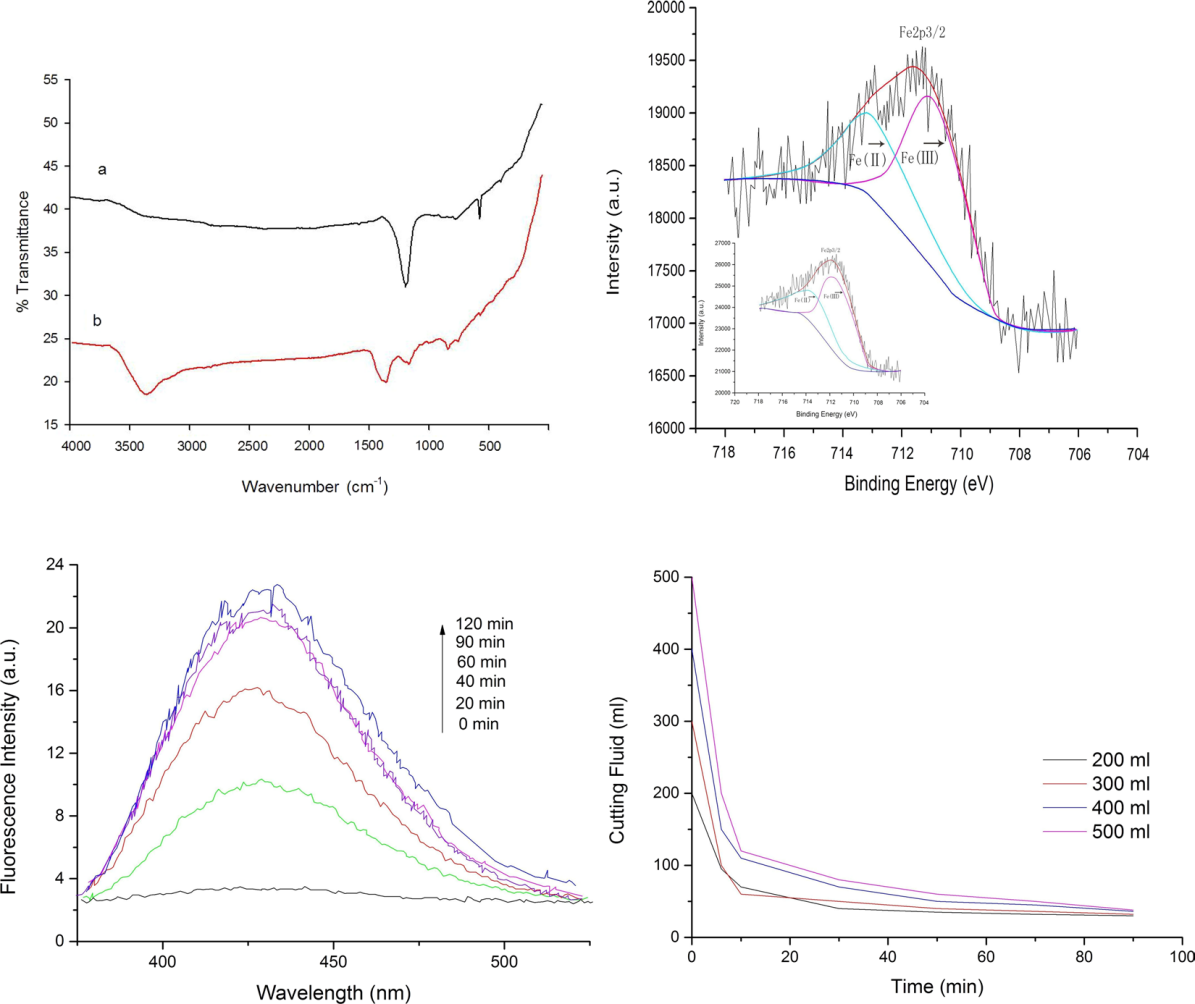
(**a**) The FT-IR spectra of the original sample and the sample recycled five times, (**b**) XPS spectra of NZVI@ACF, (**c**) Photoluminescence spectra of HTA solution, (**d**) Effects of different initial concentrations on the rate of COD removal.

In order to investigate the oxidation states on the surface of the nZVI/R composite, XPS technique was used. Figure 6b shows XPS spectra of NZVI@ACF. The analysis indicated an increase in iron content was due to some of the iron inside the material were transferred to the surface during the process of reflection. With the loss of iron on the surface, the migration process proceeded slowly. The energy spectrum of five cycles of Fe2p3XPS peaks of the activated carbon fiber were fitted, and the ratio of the content of iron at two oxidative states were determined from the peak areas of Fe^2+^ and Fe^3+^. As a result, the relative content of Fe^3+^ could be reduced. It is possible that iron participates in the catalytic reaction in the Fenton system.

The Fig. 6c shows the fluorescence spectrum of HTA at different times. The figure shows that the ·OH significantly changed at 0 min, 20 min, 40 min, 60 min and 90 min. At 120 minutes, the intensity changed smoothly. This is similar to the change in rate of COD removal, indicating that ·OH participated in the reaction.

The degradation process was a surface reaction process. It can be seen in Fig. 6d that the rate of COD removal, amount removed and initial reaction rate increased with an increased concentration. So the reaction equation could be described using the Langmuir-Hinshelwood kinetic model (equation 1).

1 $$1/{r}_{0}=1/{k}_{1}{k}_{2}\bullet 1/{c}_{0}+1/{k}_{1}$$

where r_0_ is the initial reaction rate; c_0_ is the initial concentration of cutting fluid waste; k_1_ is the reaction rate constant; k_2_ is the adsorption coefficient.

The L-H fitting parameters of the activated carbon fiber supported nanometer Iron and nanometer Iron treatment cutting fluid waste liquid are listed in Table 2. It can be seen from the data in the table. The reaction rate constant and adsorption coefficient of activated carbon fiber supported nanometer iron to remove COD are larger than that of bare nanometer iron. It was proved that the reaction rate of removal COD was positively correlated with surface adsorption of activated carbon fiber. The degradation efficiency of organic matter is closely related to the porous structure of activated carbon fiber surface^43^. Heterogeneous Fenton catalysts are rich in micropores and small-sized transition pores. Physical adsorption plays a dominant role in the adsorption of organics.Therefore, activated carbon fiber supported nanometer Iron as a heterogeneous Ferton catalyst has a stronger adsorption capacity. In the process of degradation, activated carbon fiber is used as a carrier to disperse nanometer iron particles. At the same time, the microporous structure on activated carbon fiber has a strong adsorption effect on organic matter, resulting in an increase in the concentration of organic matter around nanometer iron. In this way, the positive phase synergy between the surface adsorption of activated carbon fiber and the catalytic reaction of nanometer iron is achieved, thus improving the degradation efficiency.

**Table 2:** L-H fitting parameters for treating organic matter in NZVI@ACF and NZVI.

Treatment	k_1_ (mmol·(L·min)^−1^)	k_2_ (L·mmol^−1^)	R^2^
NZVI@ACF	0.401	7.535	0.997
NZVI	0.279	1.815	0.989

### Reaction mechanism

The mechanisms of oxidation of organic pollutants using the heterogeneous and traditional Fenton systems are similar. Under acidic conditions, the surface of NZVI generates Fe^2+^ ions and hydrogen (equation 2). Fe^2+^ and H_2_O_2_ through the Fenton oxidation process produces hydroxyl radicals that oxidize organic pollutants (equation 3). The Fe^3+^ formed in the Fenton reaction can be reduced to Fe^2+^ on the surface of NZVI, which further supports the Fenton reaction process (equation 4). At the same time H_2_O_2_ is decomposed (equation 5).

2 $${{\rm{Fe}}}^{{\rm{0}}}+{{\rm{2H}}}^{+}\to {{\rm{Fe}}}^{2+}+{{\rm{H}}}_{{\rm{2}}}$$

3 $${{\rm{Fe}}}^{2+}+{{\rm{H}}}_{{\rm{2}}}{{\rm{O}}}_{{\rm{2}}}+{{\rm{H}}}^{+}\to {{\rm{Fe}}}^{3+}+\cdot \mathrm{OH}$$

4 $${{\rm{2Fe}}}^{3+}+{{\rm{Fe}}}^{{\rm{0}}}\to {{\rm{3Fe}}}^{2+}$$

5 $${{\rm{H}}}_{{\rm{2}}}{{\rm{O}}}_{{\rm{2}}}\to {{\rm{H}}}_{{\rm{2}}}{\rm{O}}+{{\rm{O}}}_{{\rm{2}}}$$

NZVI can be passivated and forms a passivation layer on the surface in aerobic water, forming a core-shell structure. Depending on the pH of the solution, Fe_3_O_4_, Fe(OH)_2_ and other iron oxides can be formed in the shell. The Fe(OH)_2_ can be oxidized to Fe_3_O_4_ (equation 6), which can further react with dissolved oxygen in the water to form hydroxy iron oxide FeOOH (equations 3–9).

6 $${{\rm{6Fe}}}^{2+}+{{\rm{O}}}_{{\rm{2}}}+{{\rm{6H}}}_{{\rm{2}}}{\rm{O}}\to {{\rm{2Fe}}}_{{\rm{3}}}|{{\rm{O}}}_{4({\rm{s}})}+{{\rm{12H}}}^{+}$$

7 $${{\rm{Fe}}}^{2+}+{{\rm{2OH}}}^{-}\to {\rm{Fe}}{({\rm{OH}})}_{2({\rm{s}})}$$

8 $${\rm{6Fe}}{({\rm{OH}})}_{2({\rm{s}})}+{{\rm{O}}}_{{\rm{2}}}\to {{\rm{2Fe}}}_{{\rm{3}}}{{\rm{O}}}_{{\rm{4}}}+{{\rm{6H}}}_{{\rm{2}}}{\rm{O}}$$

9 $${{\rm{4Fe}}}_{{\rm{3}}}{{\rm{O}}}_{{\rm{4}}}+{{\rm{O}}}_{{\rm{2}}}\,{+\mathrm{6H}}_{{\rm{2}}}{\rm{O}}\to {{\rm{12FeOOH}}}_{({\rm{s}})}$$

In the NZVI@ACF/H_2_O_2_ system, when the pH is low, the passivation layer is thinner and H_2_O_2_ is relatively stable, promoting the formation of Fe^2+^, ·OH and increasing the rate of the oxidation reaction. When the pH is high, the passivation layer thickens and Fe_3_O_4_ and Fe(OH)_2_ predominate, resulting in NZVI@ACF passivation and loss of catalytic capacity. In addition, the ineffective decomposition of H_2_O_2_ ultimately leads to a reduction in the production of ·OH.

**Figure 7:**
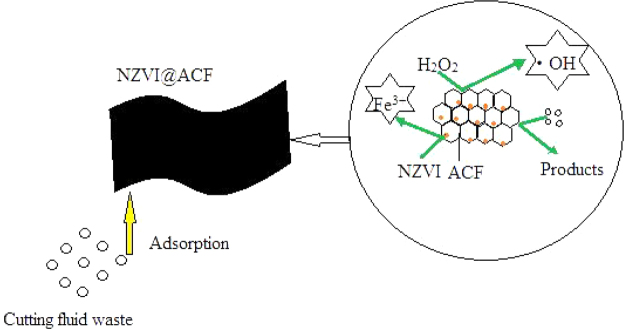
Reaction mechanism diagram.

Oxidation is not the only mechanism for the removal of pollutants using the NZVI@ACF/H_2_O_2_ system. The adsorption by ACF further increases the efficiency of the system. Figure 7 shows the reaction mechanism diagram.

## Conclusions

1. Cutting waste fluid was treated using a multi-stage chemical pretreatment-NZVI@ACF/H_2_O_2_ system. The optimum pretreatment conditions for primary flocculation included PAC (10%, 90 mL/L) and PAM (0.3%, 10 mL/L). The rate of COD removal was 79% after flocculation and demulsification. Following secondary flocculation using PFS (0.3%) at pH 7, the rate of COD removal was 85%. After the multi-stage chemical pretreatment, the optimal conditions were determined to be pH 5, H_2_O_2_ concentration 20 mmol/L and NZVI@ACF concentration 6 g/L, and the rate of COD removal was 99.8%, with the treated liquid meeting China’s tertiary wastewater discharge standards.
2. The heterogeneous Fenton catalyst (NZVI@ACF) effectively activated H_2_O_2_ to catalyze the degradation of cutting fluid under neutral pH conditions, with a rate of COD removal up to 82% after five repetitions. High catalytic activity was achieved across a pH range of 2–12, effectively increasing the traditional Fenton reaction pH range and improving the efficiency of the treatment of cutting fluid waste in industrial settings.

## Methods

### Waste water samples

Cutting fluid waste liquid samples were taken from the Qingdao Machinery company. Prior to use, the liquid was transparent, brown and did not any impurities. After two years of use, the color was a milky yellow due to the large amount of organic matter, suspended solids and other contaminants. Table 3 shows the characteristics of cutting fluid waste and China’s tertiary wastewater discharge standards.

**Table 3:** Characterization of the machining cutting fluid waste liquid and the permitted discharge limits.

Parameter	pH	Suspended solids (mg/L)	COD (mg/L)	BOD (mg/L)	P (mg/L)	NH_3_-N (mg/L)
Waste	9.96	15000	195000	26600	8000	1260
Permissible	6–9	400	500	300	5	35

### Reagents and chemicals

Polymeric ferric sulfate (PFS, 10%), polyaluminium chloride (PAC, 10%) and polyacrylamide (PAM, 0.3%) were used for multi-stage chemical pretreatment of NZVI@ACF. For the preparation of the heterogeneous Fenton system, reagents were palm fibers, sulfuric acid (H_2_SO_4_, 1 g/L), sodium hydroxide (NaOH, 6 g/L), sodium borohydride (NaBH_4_), ferric chloride hexahydrate (FeCl_3_·6H_2_O, 0.2 mol/L), and ferrous sulfate heptahydrate (FeSO_4_·7H_2_O, 0.1 mol/L). The pH was adjusted using H_2_SO_4_ (50%) and NaOH (10%).

### Preparation of Heterogeneous Fenton catalysts

**Figure 8:**
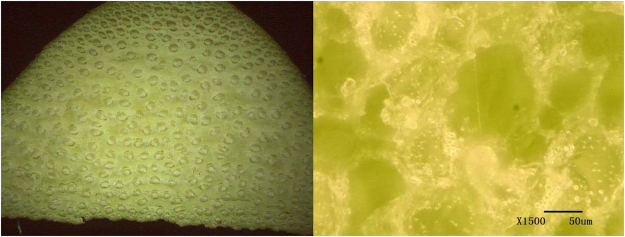
Topical view and cross-sectional view of palm fibers.

Palm fiber is composed of a large number of closely arranged fibrous cells, resulting in a highly elastic and tough fiber with an average length of 20 cm. Figure 8 shows topical and cross-sectional views of palm fiber. These images show a bundle of tubular cavities arranged in parallel with cellular features, and a rich porous structure. The center pore of the section was significantly larger than those in the surrounding areas. Each tube bundle was tightly arranged with a slit in the middle.

Dip-molding was used to make the NZVI@ACF using four steps:

1. The palm fiber was washed three times with distilled water to remove impurities remaining on the fibers, dried at 100^o^C, then soaked in H_2_SO_4_ (1 g/L) for 3 ho at room temperature, washed to neutral pH and soaked in NaOH solution (6 g/L) at 80 ^o^C for 5 h.
2. The treated fibers were washed with water until a neutral pH was obtained and immersed in an iron solution (0.35 mol/L) at room temperature for 24 h.
3. In a nitrogen atmosphere (25 mL/min), an aqueous solution of NaBH_4_ (0.15 g in 45 mL) was added to the fiber-iron solution. The resulting mixture was stirred for 60 minutes and the treated fibers washed three times with distilled water.
4. The treated fibers were carbonized in a resistance furnace for a set temperature and time under a nitrogen atmosphere to generate NZVI@ACF.

### Multistage chemical pretreatment - NZVI@ACF/H_2_O_2_ system

The optimal reagents and operating conditions of the multistage chemical pretreatment of NZVI@ACF were determined using a test tube device and PAC, PFS and PAM in different combinations to treat the cutting fluid wastewater. Change in COD was measured to determine the optimal combination. The process consisted of three steps:

1. The pH of the wastewater sample was adjusted using H_2_SO_4_ (50%, 1 L).
2. The appropriate volume of flocculent (PAC, PFS or PAC + PFS) was added to the mixture.
3. The mixture was stirred at 300 r/min for 1 min, and then at 150 r/min for 5 min. The flocculent was added slowly and the mixture stirred at 150 r/min for 3 min. The COD of the static filtered liquid was then measured.

The optimum conditions for addition of the flocculent, pH and reaction temperature were determined using a single factor test. The wastewater was treated with the heterogeneous Fenton system NZVI@ACF/H_2_O_2_. The effects on pH v, quantity of catalyst, H_2_O_2_ input and reaction time were determined and the optimal value of each factor was found.

### COD rate of removal rate and heterogeneous Fenton catalyst analysis method

The rapid closed digestion method was used to determine a decrease in COD following the treatment of cutting fluid waste water using the heterogeneous Fenton fibre system. The rate of COD removal was tested using the heterogeneous Fenton catalyst at various pH, different amounts of catalyst and H_2_O_2_, and the results were compared with treatment using the traditional Fenton catalyst.

The iron was successfully attached to the active carbon fiber. This was analyzed using X-ray diffraction, adsorption of nitrogen adsorption isotherm and SEM analysis.
